# Supplementary material for: Global burden and trends of age-related and other hearing loss: A 32-year analysis and future projections based on the GBD 2021
Source: PLoS One. 2025 Aug 21;20(8):e0330690. doi: 10.1371/journal.pone.0330690 (PMC12370029; doi:10.1371/journal.pone.0330690)
Supplement: S1 File — S1 Fig. Global and regional trends in the ASPR and ASYR for ARoHL, 1990–2021. This figure illustrates the temporal trends in the ASPR and ASYR for ARoHL globally and across five SDI regions from 1990–2021, stratified by sex. These trends highlight the differential impact of ARoHL on various populations and underscore the importance of sex-specific analyses in understanding disease burden. (A) ASPR in both sexes; (B) ASPR in females; (C) ASPR in males; (D) ASYR in both sexes; (E) ASYR in females; (F) ASYR in males. S2 Fig. Correlation between the SDI and the ASPR/ASYR for ARoHL across 204 countries and territories in 2021. This figure illustrates the correlation between the SDI and the ASPR/ASYR for ARoHL across 204 countries and territories in 2021. This figure highlights the relationship between the SDI and the ARoHL burden, with scatter plots showing trends and correlations. Different colored dots represent different countries or territories. The gray curve represents the relationship between the SDI and the metric, with the shaded area indicating the 95% confidence interval. (A) SDI vs. ASPR; (B) SDI vs. ASYR. S3 Fig. Relationships between the EAPC and the ASPR/ASYR/SDI across 204 countries and territories, 1990–2021. This figure illustrates the relationships between the EAPCs (1990–2021) and the ASPR, ASYR, and SDI across 204 countries and territories. It includes scatter plots with regression lines and corresponding R and P values, indicating the temporal trends and sociodemographic influences on the ARoHL burden. Each dot represents a country or territory, with the size of the dot indicating the magnitude of the value. The blue curve shows the relationship between the X-axis and Y-axis parameters, with the shaded area representing the 95% confidence interval. (A) ASPR vs. its EAPC; (B) SDI vs. EAPC for ASPR; (C) ASYR vs. its EAPC; (D) SDI vs. EAPC for ASYR. S4 Fig. Frontier analysis. This figure identifies countries with the highest potential for improvement i [file pone.0330690.s001.zip › Supporting information/S1_Table.docx]

**S1 Table. Age-standardized prevalence rate (ASPR) per 100,000 for age-related and other hearing loss in 1990 and 2021 and its estimated annual percentage change (EAPC) from 1990**–**2021 at the global and regional levels.**

|  | **1990** | | **2021** | | **1990-2021** | |
| --- | --- | --- | --- | --- | --- | --- |
|  | **Prevalent cases,**  **(95% UI)** | **ASPRs**  **per 100 000**  **(95% UI)** | **Prevalent cases,**  **(95% UI)** | **ASPRs**  **per 100 000**  **(95% UI)** | **Total percent change**  **(95% UI)** | **EAPC, %,**  **(95% CI)** |
| Global | 7.41 (7.06, 7.80) ×10^8^ | 1.71 (1.63,1.80) ×10⁴ | 1.55 (1.48, 1.62) ×10^9^ | 1.81 (1.73, 1.89) ×10^4^ | 1.09(1.06,1.11) | 0.163(0.154,0.172) |
| SDI |  |  |  |  |  |  |
| High | 1.77 (1.68, 1.86) ×10^8^ | 1.38 (1.32,1.45) ×10⁴ | 2.50 (2.39, 2.63) ×10^8^ | 1.41 (1.35, 1.48) ×10^4^ | 0.72(0.7,0.73) | 0.058(0.048,0.069) |
| High-middle | 2.35 (2.24, 2.49) ×10^8^ | 1.71 (1.63,1.80) ×10⁴ | 3.41 (3.25, 3.58) ×10^8^ | 1.86 (1.78, 1.95) ×10^4^ | 0.93(0.89,0.97) | 0.279(0.271,0.287) |
| Middle | 1.33 (1.26, 1.40) ×10^8^ | 1.91 (1.82,2.02) ×10⁴ | 5.40 (5.15, 5.68) ×10^8^ | 1.97 (1.89, 2.07) ×10^4^ | 1.3(1.25,1.35) | 0.086(0.075,0.097) |
| Low-middle | 4.96 (4.68, 5.26) ×10^7^ | 1.79 (1.70,1.88) ×10⁴ | 2.95 (2.82, 3.10) ×10^8^ | 1.81 (1.74, 1.90) ×10^4^ | 1.22(1.18,1.25) | 0.009(-0.006,0.023) |
| Low | 1.77 (1.68, 1.86) ×10^8^ | 1.73 (1.65,1.82) ×10⁴ | 1.18 (1.12, 1.25) ×10^8^ | 1.76 (1.68, 1.85) ×10^4^ | 1.38(1.37,1.4) | 0.039(0.012,0.065) |
| Regions |  |  |  |  |  |  |
| Andean Latin America | 3.48 (3.30, 3.67) ×10^6^ | 1.43 (1.36,1.50) ×10⁴ | 8.98 (8.56, 9.45) ×10^6^ | 1.44 (1.37, 1.52) ×10^4^ | 1.58(1.54,1.63) | 0.055(0.047,0.064) |
| Australasia | 3.20 (3.09, 3.32) ×10^6^ | 1.39 (1.35,1.45) ×10⁴ | 6.78 (6.43, 7.18) ×10^6^ | 1.43 (1.36, 1.51) ×10^4^ | 1.12(1.03,1.21) | 0.144(0.108,0.180) |
| Caribbean | 4.36 (4.14, 4.61) ×10^6^ | 1.55 (1.48,1.63) ×10⁴ | 8.18 (7.79, 8.62) ×10^6^ | 1.55 (1.48, 1.64) ×10^4^ | 0.88(0.85,0.9) | 0.011(0.007,0.015) |
| Central Asia | 8.23 (7.82, 8.66) ×10^6^ | 1.59 (1.51,1.67) ×10⁴ | 1.42 (1.35, 1.49) ×10^7^ | 1.60 (1.53, 1.68) ×10^4^ | 0.73(0.71,0.75) | 0.042(0.027,0.057) |
| Central Europe | 2.32 (2.21, 2.44) ×10^6^ | 1.61 (1.54,1.69) ×10⁴ | 3.02 (2.87, 3.19) ×10^7^ | 1.63 (1.56, 1.71) ×10^4^ | 0.3(0.28,0.32) | 0.047(0.043,0.051) |
| Central Latin America | 1.59 (1.51, 1.68) ×10^7^ | 1.57 (1.49,1.65) ×10⁴ | 4.05 (3.86, 4.26) ×10^7^ | 1.57 (1.50, 1.65) ×10^4^ | 1.54(1.5,1.59) | 0.015(0.012,0.018) |
| Central Sub-Saharan Africa | 4.80 (4.51, 5.10) ×10^6^ | 1.57 (1.50,1.65) ×10⁴ | 1.23 (1.15, 1.31) ×10^7^ | 1.55 (1.48, 1.63) ×10^4^ | 1.56(1.51,1.6) | -0.026(-0.046,-0.006) |
| East Asia | 2.04 (1.94, 2.18) ×10^8^ | 2.06 (1.95,2.19) ×10⁴ | 4.59 (4.34, 4.86) ×10^8^ | 2.19 (2.08, 2.31) ×10^4^ | 1.24(1.18,1.31) | 0.203(0.185,0.222) |
| Eastern Europe | 4.34 (4.14, 4.57) ×10^7^ | 1.62 (1.54,1.70) ×10⁴ | 5.05 (4.81, 5.32) ×10^7^ | 1.63 (1.55, 1.71) ×10^4^ | 0.16(0.15,0.18) | 0.028(0.018,0.039) |
| Eastern Sub-Saharan Africa | 1.99 (1.87, 2.12) ×10^7^ | 1.99 (1.88,2.11) ×10⁴ | 5.01 (4.72, 5.34) ×10^7^ | 2.08 (1.96, 2.22) ×10^4^ | 1.52(1.49,1.55) | 0.150(0.053,0.248) |
| High-income Asia Pacific | 2.78 (2.65, 2.92) ×10^7^ | 1.39 (1.33,1.46) ×10⁴ | 5.06 (4.82, 5.33) ×10^7^ | 1.41 (1.34, 1.47) ×10^4^ | 0.82(0.78,0.86) | 0.035(0.029,0.040) |
| High-income North America | 4.75 (4.49, 5.03) ×10^7^ | 1.41 (1.34,1.49) ×10⁴ | 7.99 (7.57, 8.50) ×10^7^ | 1.37 (1.30, 1.44) ×10^4^ | 0.68(0.65,0.72) | -0.150(-0.189,-0.112) |
| North Africa and Middle East | 2.97 (2.80, 3.13) ×10^7^ | 1.45 (1.38,1.52) ×10⁴ | 7.60 (7.25, 8.00) ×10^7^ | 1.46 (1.39, 1.53) ×10^4^ | 1.56(1.52,1.6) | 0.032(0.029,0.036) |
| Oceania | 7.43 (7.04, 7.88) ×10^5^ | 1.94 (1.85,2.05) ×10⁴ | 1.87 (1.78, 1.98) ×10^6^ | 1.97 (1.87, 2.09) ×10^4^ | 1.52(1.48,1.56) | -0.026(-0.051,-0.001) |
| South Asia | 1.33 (1.25, 1.41) ×10^8^ | 1.85 (1.77,1.95) ×10⁴ | 3.15 (3.01, 3.30) ×10^8^ | 1.90 (1.82, 1.99) ×10^4^ | 1.37(1.32,1.42) | 0.024(0.002,0.047) |
| Southeast Asia | 6.39 (6.07, 6.75) ×10^7^ | 2.04 (1.94,2.15) ×10⁴ | 1.48 (1.41, 1.56) ×10^8^ | 2.09 (1.99, 2.20) ×10^4^ | 1.31(1.27,1.36) | 0.020(0.000,0.041) |
| Southern Latin America | 6.13 (5.82, 6.46) ×10^6^ | 1.31 (1.25,1.38) ×10⁴ | 1.10 (1.04, 1.16) ×10^7^ | 1.32 (1.26, 1.40) ×10^4^ | 0.79(0.76,0.82) | 0.031(0.027,0.034) |
| Southern Sub-Saharan Africa | 5.42 (5.18, 5.69) ×10^6^ | 1.58 (1.52,1.65) ×10⁴ | 1.11 (1.06, 1.16) ×10^7^ | 1.61 (1.54, 1.68) ×10^4^ | 1.04(1.01,1.07) | 0.077(0.054,0.100) |
| Tropical Latin America | 1.90 (1.80, 2.01) ×10^7^ | 1.76 (1.67,1.86) ×10⁴ | 4.54 (4.32, 4.79) ×10^7^ | 1.77 (1.68, 1.86) ×10^4^ | 1.39(1.35,1.44) | 0.008(-0.014,0.030) |
| Western Europe | 6.15 (5.86, 6.46) ×10^7^ | 1.18 (1.13,1.24) ×10⁴ | 8.91 (8.51, 9.35) ×10^7^ | 1.19 (1.14, 1.25) ×10^4^ | 0.45(0.44,0.46) | 0.008(0.001,0.016) |
| Western Sub-Saharan Africa | 1.53 (1.43, 1.63) ×10^7^ | 1.38 (1.30, 1.45) ×10⁴ | 3.72 (3.46, 3.99) ×10^7^ | 1.34 (1.27, 1.41) ×10^4^ | 1.43(1.41,1.46) | -0.054(-0.108,-0.001) |

ASPR = age-standardized prevalence rate; EAPC = estimated annual percentage change; SDI = sociodemographic index;

95% UI = 95% uncertainty interval; 95% CI = 95% confidence interval.
