# Supplementary material for: Global burden and trends of age-related and other hearing loss: A 32-year analysis and future projections based on the GBD 2021
Source: PLoS One. 2025 Aug 21;20(8):e0330690. doi: 10.1371/journal.pone.0330690 (PMC12370029; doi:10.1371/journal.pone.0330690)
Supplement: Attachment — Submitted filename: Response to Reviewers.docx [file pone.0330690.s003.docx]

We sincerely thank the reviewers for their careful review of our manuscript and for providing valuable and constructive feedback. We have carefully addressed each comment and have also conducted a thorough self-review, making further revisions to enhance the quality of our manuscript. In addition, the language of the entire paper has been polished by AJE.

We have used Microsoft Word’s “Track Changes” feature to document the specific revisions. In the attached manuscript, some extensive modifications are highlighted in yellow for your quick reference. A detailed summary of our responses to your comments and the revisions we have made is provided in the table below.

We believe that these revisions have significantly improved the clarity and quality of our manuscript. We appreciate your time and consideration and look forward to your positive response.

1. **Reviewers' comments （Reviewer #1）**

| **Reviewers' comments** | **Response** |
| --- | --- |
| 1. Methods: The study utilized a variety of instruments such as the health inequality analysis. Based on the populations of countries like China and India, their contribution which had the largest impact on Slope index of Inequality, might have been due this factor. Hence comparism with smaller countries may not truly give realistic values. Hence might tend to have inherent sampling bias. | **We acknowledge the limitation regarding the influence of large-population countries on the Slope Index of Inequality. Unfortunately, there is no universally accepted method to fully address this issue** |
| 2. References: The referencing style appears generally fine. However, it appears the reference cited (references 9) due to its publication date, 1978 might not be reflective of current trends due to being slightly outdated. | **We have replaced the outdated reference (Reference 10) with a more recent and relevant source to better reflect current trends** |
| 3. The phrasing of the language used in the entire document could benefit from a more professional grammatical input. | **Taking your suggestions into consideration, we have had the language of the entire document polished by AJE.** |
| 4. I found the title, study design, statistics, figures and tables quite satisfactory. | **We appreciate the reviewer's positive feedback on the title, study design, statistics, figures, and tables. However, after revising and polishing the entire manuscript, we have made minor adjustments to these sections to ensure overall consistency.** |

2. **Reviewers' comments （Reviewer #2）**

| **Reviewers' comments** | **Response** |
| --- | --- |
| **General:** |  |
| 1) Provide a table with definitions for all acronyms used throughout the manuscript. | **In response to your suggestion, a “List of Abbreviations” has been added immediately following the Conclusion section. This table provides definitions for all acronyms and abbreviations used throughout the manuscript to enhance clarity.** |
| 2) When giving numbers, show only the significant digits and use exponential notation when spanning several orders of magnitude. Do this throughout the text and also for Table 1 (which is difficult to read as it is). | **We have revised the numerical data presentation throughout the text to show only significant digits and used exponential notation where appropriate. Additionally, Table 1 has been revised and moved to the Supporting Information as S1 Table. We have also applied the same formatting changes to S2 Table.** |
| 3) Throughout the text, as a “standard”, state and use and discuss all variables in terms of normalized (for population size; i.e., per 100,000). When stating/discussion “raw”, un-normalized values, then clearly state that (and distinguish from normalized values). | **We have standardized the presentation of all variables throughout the manuscript using normalized values (per 100,000) according to this suggestion. For ASPR and ASYR, we have explicitly noted that these are expressed per 100,000. Additionally, we have clearly distinguished raw, un-normalized values by specifying them as “number of cases” or “number of YLDs” whenever they appear. This ensures consistency and clarity in our data presentation.** |
| **Specific:** |  |
| 1) The reference list is difficult to read. Re-format. For example, for a given reference indent all lines after the first. In addition, double space between references. | **We have reformatted the references in accordance with your request.** |
| 2) Table 1 is difficult to read. Re-format per above. Consider putting this into supplementary material (and relying on the figures). | **We have re-formatted Table 1 as requested and renamed it as S1 Table, placing it in the Supporting Information.** |
| **3) Abstract** |  |
| a. Emphasize prevalence and YLDs that are normalized for population size (e.g., cases per fixed number of people within a given population). | **In the abstract, we have explicitly noted that ASPR and ASYR are expressed per 100,000, emphasizing the use of normalized values as requested.** |
| b. Emphasize trends, disparities, and differences between using normalized values (for population size) for prevalence an YLDs. | **We have revised the abstract to emphasize the trends, disparities, and differences in prevalence and YLDs when using normalized values (per 100,000).** |
| 4) Add more detail, emphasis, and discussion about normalizing the prevalence, YLDs, and distribution of ARoHL with respect to the population size at each time point being analyzed. In other words, prevalence per fixed number of people within a given population. The normalized variables are more meaningful in terms of explaining possible effects of the various “driving” factors (compared to the effect of increases/decreases in the total population size on the raw, absolute numbers). | **We have revised the manuscript to emphasize and discuss the normalization of prevalence, YLDs, and ARoHL distribution with respect to population size at each time point, focusing primarily on the normalized variables as requested.** |
| 5) Provide some examples of possible actions that could be taken and strategies that could be proposed, in view of the projections for future trends. | **We have incorporated examples of possible actions and strategies that could be proposed based on the projections for future trends, as requested.** |
| **6) Decomposition Analysis** |  |
| a. Discuss possible other/confounding factors, including the following: noise-induced hearing loss (outside environment and home/work conditions), side-effects of medical treatments (medications/drugs, surgery, radiation), family history and genetic pre-dispositions. | **We have added a discussion of additional potential confounding factors, including noise-induced hearing loss, medical treatment side effects, and genetic predispositions, in the discussion section.** |
| b. Line 134: Provide a table listing all the factors “behind changes in the disease burden of ARoHL” and their percent contributions. Emphasize the percent contributions for changes in a normalized (for population size) prevalence of ARoHL. | **We have detailed the drivers of absolute changes in ARoHL burden in Supplementary Table S5, quantifying contributions from population growth, aging, and epidemiological shifts. For normalized prevalence (ASPR), given its design to remove population size effects, we currently believe that attributing specific drivers would be methodologically unsound. Instead, we have analyzed the relationships between ASPR and factors such as age, gender, and SDI throughout the text.** |
| c. Line 140: Discuss “the accelerated aging process”. | **We apologize for the oversight in our manuscript where we inadvertently used the term “the accelerated aging process” instead of “population aging.” We have now corrected this error. While we acknowledge the importance of discussing population aging, we did not elaborate further on this topic in our discussion, as population aging is difficult to change in the short term, as mentioned in our discussion section.** |
| d. Line 147: Explain and discuss the meaning of “for a comprehensive understanding of the epidemiological evolution of middle ear disease”, within the context of ARoHL. Why refer to “middle ear disease”? | **We appreciate the reviewer’s attention to this point. The reference to “middle ear disease” was indeed an oversight, and we intended to discuss ARoHL. We were concurrently working on a study related to middle ear disease, which likely contributed to the confusion. We have now corrected this error and have thoroughly reviewed the entire manuscript to ensure consistency and accuracy, minimizing the possibility of similar mistakes.** |
| **7) Results** |  |
| a. Emphasize ASPR per 100,000 people within a given population (i.e., normalized for population size). | **We have made the revision as requested.** |
| b. Lines 216 - 218: State that these numbers for prevalence are a raw number of cases in the population. State that the ASPR is per 100,000 (i.e., normalized for population size. It is especially meaningful to emphasize changes in a normalized prevalence (cases per fixed number of people within a population) over the 21-year time period. Thereafter, one can consider possible factors involved (other than population increase or decrease). | **We have made the revision as requested.** |
| c. Lines 218 – 219: In regard to “age-standardized prevalence rate (ASPR)”, provide a literature reference/citation that details/describes comparing across populations with different age structures (using weights based on the age distribution of a standard population). | **We have addressed this point by providing a literature reference and detailed explanation regarding the calculation of the age-standardized prevalence rate (ASPR) and age-standardized YLDs rate (ASYR) in the “Estimation of disease burden” section of the Materials & Methods.** |
| d. Table 1 legend: State the EAPC is based on the normalized ASPRs (per 100,000 people). | **We have made the revision as requested.** |
| e. Figure 1 legend: State normalized ASPR and normalized YLDs are shown in the figure. | **We have made the revision as requested.** |
| **8) Conclusions** |  |
| a. Lines 448 – 452: Expand. Besides “raw”, un-normalized total numbers, emphasize changes in numbers that are normalized for population size. Summarize the percent changes (for normalized data). This way one can meaningfully consider changes in prevalence related to all factors (other than population growth) which might be important for considering intervention strategies. Finally, suggest (speculate) possible intervention strategies. | **We have made the revision as requested.** |
